# Assessment of mood after severe acquired brain injury: Interviews with UK clinical psychologists and medical professionals

**DOI:** 10.1177/02692155241278289

**Published:** 2024-09-10

**Authors:** Alexandra E Rose, Breda Cullen, Sarah Crawford, Jonathan J Evans

**Affiliations:** 1 School of Health and Wellbeing, College of Medical, Veterinary and Life Sciences, University of Glasgow, Glasgow, UK; 2 59386 Royal Hospital for Neuro-disability, London, UK

**Keywords:** Brain injury, mood, assessment, qualitative study, clinical evaluation

## Abstract

**Objective:**

Mood assessment is challenging when people have cognitive and receptive communication impairments after severe brain injury. This study explored how UK-based medical and psychology professionals working with people with severe cognitive and communication impairments after brain injury assess mood in this population.

**Design:**

Following their participation in an online survey, professionals were invited to participate in individual semi-structured interviews. Interviews were analysed using thematic analysis to label explicit data (semantic themes) and implicit data (latent themes).

**Participants:**

Twenty-three clinical psychologists and nine medical professionals participated in online or in-person interviews.

**Results:**

Both groups explicitly reported using a combination of collateral information, history, observations, and patient interviews when assessing mood in this population. Medical professionals did not routinely use standardised measures and clinical psychologists often adjusted them when they used them. The respondents discussed difficulties conceptualising depression after brain injury, the experience needed by the assessor, and the need for an individualised approach for this population. Clinical psychologists discussed the pressures of working in healthcare systems and medical professionals discussed how symptoms may influence prescription choices. Seven latent themes were labelled which highlighted additional challenges and complexities experienced by those assessing mood, beyond the actual assessment process itself.

**Conclusions:**

No ‘gold standard’ approach to assessing mood in those with cognitive and communication difficulties after severe brain injury was identified. There was overlap in assessment approaches but no clear consensus. Interviewees felt that mood assessment must be approached differently in this population and that self-report measures are not useful.

## Introduction

Acquired brain injury (ABI) refers to damage to the brain sustained since birth through a variety of causes including traumatic events (e.g. falls, car accidents, assaults), infection, tumour, strokes or hypoxia (e.g. following cardiac arrest or carbon monoxide poisoning).^1^ Health professionals in the United Kingdom are advised to screen for the presence of mood difficulties following ABI by national clinical guidelines.^2–7^ A recent systematic review^8^ showed that patients with severe brain injuries have generally been excluded from validation studies of standardised mood measures. When the small number of studies that have included these patients were examined, it was found that no self-reported mood measures could be recommended for use with this population. Whilst this potentially makes observer-rating scales more attractive, these are also problematic.

Neurorehabilitation teams are advised to be (medical) consultant led^6^ Medical professionals are therefore ultimately responsible for the treatment of the individuals in their care. The guidelines on antidepressant use after brain injury suggest asking about mood in each clinical interview completed by the medical team. In those where depression is identified, guidelines suggest that members of the multidisciplinary team (MDT) should measure severity using standardised measures. When there is a complex presentation regarding mood, recommendations suggest that this should be conducted by a clinical psychologist (Appendix 2, p. 16, RCP, 2005^2^).

Current UK national guidelines^2–7^ address the issue of how to assess mood in patients with communication impairments, but do not give clear guidance on assessing those with severe cognitive impairments which preclude their ability to complete a clinical interview. Severe brain injury can result in complex medical and physical consequences which can obscure mood symptoms and make assessment more challenging.^9,10^ Given the complexity of patients with severe cognitive impairments and communication difficulties, a consensus on how to assess mood in this population would be beneficial to ensure timely diagnosis and treatment.

Prior to participation in the interviews reported in this study, respondents participated in a survey of professionals undertaken by the authors (under peer review) which showed there was no gold standard approach when assessing this population. However, the format of a survey limited the details of responses provided to questions. Therefore, the aim of this study was to obtain more detailed information on how those responsible for assessing mood after brain injury (i.e. clinical psychologists and medical professionals) assess this population in routine clinical practice, to establish if a professional consensus is evident. Semi-structured interviews were utilised to allow respondents to previous surveys to expand upon their survey answers and provide richer detail on:

- Their views on the assessment of mood after severe brain injury;
- Which assessment tools they use and how they use them;
- Which symptoms they assess;
- How they differentiate between mood and cognitive symptoms and
- How prescription choices are made (medical professionals).

## Method

### Ethics

Ethical approval was granted by the University of Glasgow College of Medical, Veterinary and Life Sciences Ethics Committee for Non-Clinical Research Involving Human Participants (Applications 200190181, 200200087).

### Procedure

Purposive sampling was employed via respondents’ participation in an online survey by the authors (under peer review) on assessing mood in the patient population of interest. The final survey question asked participants to indicate if they would be willing to complete a semi-structured interview about their clinical practice.

An interview protocol was created which asked participants to provide details regarding their training and experience, and answer questions regarding how they assess mood in this population. Within the protocol, a paragraph describing the patient population was designed to increase confidence that participants were discussing the same patient population.

Interviews were arranged and recorded using online platform recording (Zoom) and/or a voice recorder for transcription and thematic analysis. The study aimed to interview 15 or more participants per group in order to establish saturation.^11^

Interviews were completed by a female qualified clinical psychologist (AR) with an additional postgraduate diploma in applied neuropsychology and nearly 10 years of working in the field of acquired brain injury. The study was completed as part of her PhD in assessing mood after severe acquired brain injury. Respondents were aware the interviews were a part of the PhD study. As this field is small due to the specialisation, it was expected that AR may have worked with or know some of the respondents and the use of a semi-structured interview protocol was used to increase the consistency of the topics explored, regardless of the relationship with the interviewee. Field notes and supervision were used to reflect on AR's position and interpretation, and revisit data accordingly. Interviews of clinical psychologists took place between November 2020 and March 2021, and medical professionals were interviewed between November 2021 and June 2022.

### Reporting

The Consolidated Criteria for Reporting Qualitative Studies (COREQ) guidance and checklist were used to report on the semi-structured interview results.^12^

### Data analysis

The interviews were analysed using reflexive thematic analysis.^13–16^ A constructionist epistemological view of the data was taken.^16^ An experiential orientation was adopted when interpreting the data, focussing on participants’ subjective experience and views of assessing mood in this population. An inductive approach was used rather than using pre-defined themes, working with the data from the bottom-up.^13^ Data was analysed for both semantic themes (which addressed the practicalities of the research questions), and latent themes which were implicit within the interviews. Interviews were analysed and labelled within the two separate groups (clinical psychologists and medical professionals) as the process used and the latent themes differed according to their experience and job role.

The principal investigator (AR) followed the six stage systematic approach suggested by Braun and Clarke^13–16^ when completing thematic analysis: familiarisation with data; coding; generating initial themes; reviewing and developing themes; refining, defining and naming themes; and writing up. This process is not linear and the expectation is that the researcher would revisit and review data multiple times.

Transcripts were coded using NVIVO software by the principal investigator (AR). Themes and sub-themes were labelled, with coded extracts of the transcriptions saved under each. The codes were collated into common ideas expressed by participants in order to identify how these themes related to each other. A thematic map was created to examine relationships and overlap.

## Results

Sixteen medical professionals and 33 clinical psychologists consented to participating in an audio recorded semi-structured interview. Following contact from the principal investigator, some respondents did not respond further or could not participate in the interview. 23 interviews were arranged with clinical psychologists and nine interviews were arranged with medical professionals. All clinical psychologist interviews took place online. Three of the medical professionals’ interviews were in person with the remaining interviews taking place online. The interviews were approximately 60 minutes long. Only the interviewer (AR) and participant were present for interviews and no repeat interviews were required.

All 33 clinical psychologists were fully qualified, with 16 having postgraduate training in neuropsychology. Thirty-one of the interviewees had direct experience of working with people with severe brain injury and persisting cognitive and communication impairments. Interviewees listed a variety of measures they would use, however, when using measures with those with severe cognitive impairments, all respondents reported they make adjustments and use measures qualitatively rather than using the scores as designed, highlighting that they are insufficient in this population. (See Table 1 in the Supplemental materials for participant experience and standardised mood measure usage.)

All 16 medical professionals were fully qualified and consisted of 5 consultant doctors in rehabilitation medicine, 2 ward-based junior doctors, 1 neuropsychiatrist and 1 psychiatrist. Only one interviewee had minimal experience with those with severe brain injury, the remaining respondents worked directly with the patient population of interest. Two participants reported using standardised mood measures (the neuropsychiatrist and one rehab medicine consultant). (See Table 2 in the Supplemental materials for participant experience and standardised mood measure usage.)

### Thematic analysis

Six semantic themes (explicit data reported within groups) and seven latent themes (implicit data) were labelled during the data analysis.

### Semantic themes

Six semantic themes were labelled which related to explicit data provided by participants’ regarding their understanding of the issue and the assessment process they use. These results are reported collectively (medical professionals and psychologists) with issues or processes highlighted by one discipline only being specified. (see Supplemental Tables 3 and 4 for a summary of the semantic themes according to each group).

#### Conceptualisation of depression

Participants discussed that, when assessing mood in this population, they are aware that some symptoms that may reflect depression in people without brain injury arise as a consequence of injury to the brain. This leads them to question whether the construct of depression should be conceptualised in the same way in people with and without severe brain injury.

There were divided views as to whether depression after brain injury is the same as depression in the non-brain injured population. There were views that depression after severe ABI may be different and this impacts the diagnostic process. Others felt that depression is the same and that the brain injury should not preclude a mood disorder diagnosis, but acknowledged a different way of assessing may be required.

When considering whether to assess for the presence of a mood disorder, clinical psychologists consider the reason for the assessment, whether what they are seeing is indicative of adjustment to the brain injury and/or the environment and whether mood is the likely cause of difficulties.

Medical professionals discussed how changes in neurotransmitters after an acquired brain injury can cause issues with mood. It was noted that depression arising from disruption to neurotransmission in the brain may be distinct from depression arising from a reaction to events or a change in a person's social situation.

#### Assessment

It was discussed that organic/medical issues can cause deterioration in functioning during recovery from brain injury but so can depression, highlighting the importance of ruling out organic causes for symptoms that may be mistaken for depression. Respondents also considered the nature of the ABI when thinking about the assessment of mood. These organic considerations alluded to the fact that prior to treating a mood disorder, medical professionals would screen for other causes of the presentation that may require a different intervention or treatment.

When discussing their approach to assessment, the interviewees in both groups would talk to the patient, keeping the language and the questions simple. Additionally they would collect collateral information from the multidisciplinary team and the family, including information on any premorbid history of mood difficulties, as well as observations of behaviours and symptoms, given that the patient may not be able to give an accurate history or communicate their experience. A careful assessment of the chronology of symptoms and monitoring over time characterises the assessment approach.

The clinical psychologists who had less experience working with people with severe persisting cognitive and communication impairments appeared to struggle to express the complexities of assessing mood in this population, often referring to clinical guidelines, which those with more experience repeatedly suggested were insufficient to guide practice with this population.

Symptoms that would be monitored included reduced or poor engagement and participation, tearfulness, emotional lability, neurovegetative symptoms (changes in sleep and appetite), poor initiation or apathy, reports of feeling sad/angry/irritable/low, suicidal ideation and poor reactivity to others/the environment. These would need to be separated from symptoms expected to be associated with the person's brain injury. Both clinical psychologists and medical professionals highlighted a formulation-based approach designed to consider multiple factors that may influence a person's presentation and come to a reasonable conclusion on how to understand the patient.

#### The person with a severe ABI

An individualised approach to assessment was favoured, including formulation. When discussing assessment, it was noted that adjustments to assessments are necessary to accommodate for cognitive impairment and communication difficulties. An additional theme was whether cognitive impairment impacts someone's ability to be depressed.

An interesting subtheme was noted where a respondent felt that being labelled as ‘brain injured’ can lead to an assumption that both assessment and treatment will be different than for people without brain injury, but the respondent viewed this assumption as inappropriate and incorrect. This comment related to less experienced clinicians feeling the need to refer patients to specialists rather than attempting to review or treat them. It also related to views that brain injured people may be vulnerable to being pathologised rather than being seen as human beings with typical human experiences, emotions and reactions. Cultural difference as a possible additional discrimination was also noted.

#### The assessor

More experienced clinical psychologists were more dismissive of standardised measures, relying more on clinical opinion (based on knowledge acquired through direct patient experience). Respondents suggested that using measures may make assessors feel less anxious as they have used something ‘structured’ to assess mood, but that they were not particularly useful as a diagnostic tool. Medical professionals also spoke of their methods of assessment and choices of medication in relation to the experience they had, with less experienced medical professionals reporting they would defer to a colleague or refer to another specialist. More experienced clinicians expressed concern that those with less experience may not have the ability to assess this population as they viewed it as specialist assessment. A number of the medical professionals noted that the symptoms of depression may be missed by those not experienced in working with brain injury, as they may not know what symptoms/indicators to look for.

#### Treatment options (medical professionals)

All medical professionals felt there was a role for medication if treatable symptoms were identified. Some also mentioned they may use medication to improve engagement even if they did not feel the person had depression. Medication choices were based on personal preference and experience, previous medications that were successful for the patient or medications that may address specific symptoms, for example, poor appetite, increased tone, poor sleep or pain. Side effects were considered and it was highlighted that medications needed review as they could cause symptoms that may cloud the assessment of mood. There was a concern from both groups of interviewees that antidepressants may not be removed once started, which related to concerns about a lack of appropriate community follow up.

In terms of monitoring treatment effects, interviewees noted the importance of monitoring for improvement in the identified symptoms specific to the person. Formal outcome measures were rarely mentioned as useful for this purpose and individualised approaches were favoured.

Other treatment options were discussed with the general view being that both pharmacological and non-pharmacological treatments may be useful. Whilst medication was not viewed as necessarily a first line of intervention, there was acknowledgement that severe cognitive and communication impairments may preclude these patients from traditional psychological or non-pharmacological interventions.

#### The system (clinical psychologists)

Many of the clinical psychologists spoke about the potential for the wider context in which assessments are taking place (e.g. the healthcare system including the multidisciplinary team, and the patient's family system) to influence their approach to assessment. It was noted that working within these systems may lead to using measures due to requirements from the service as opposed to them being viewed as appropriate. Additional systemic issues included lack of staff, fewer clinical psychologists in multidisciplinary teams, and a lack of cross-over between clinical psychologists and medical professionals resulting in reduced communication opportunities.

To summarise the current clinical practice described in these interviews, visual representations are provided in the Supplemental materials (Supplemental Figures 1 and 2).

### Latent themes

**Figure 1.**
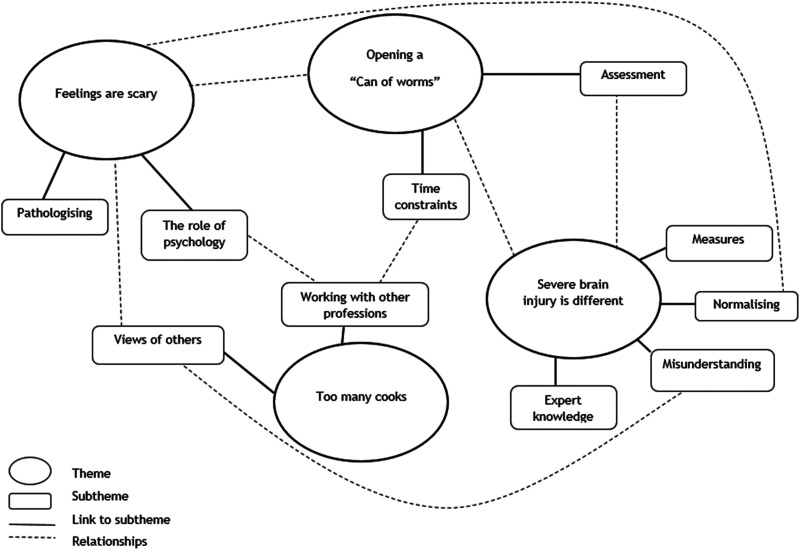
Clinical psychologist interviews: implicit themes and how they relate to each other.

**Figure 2.**
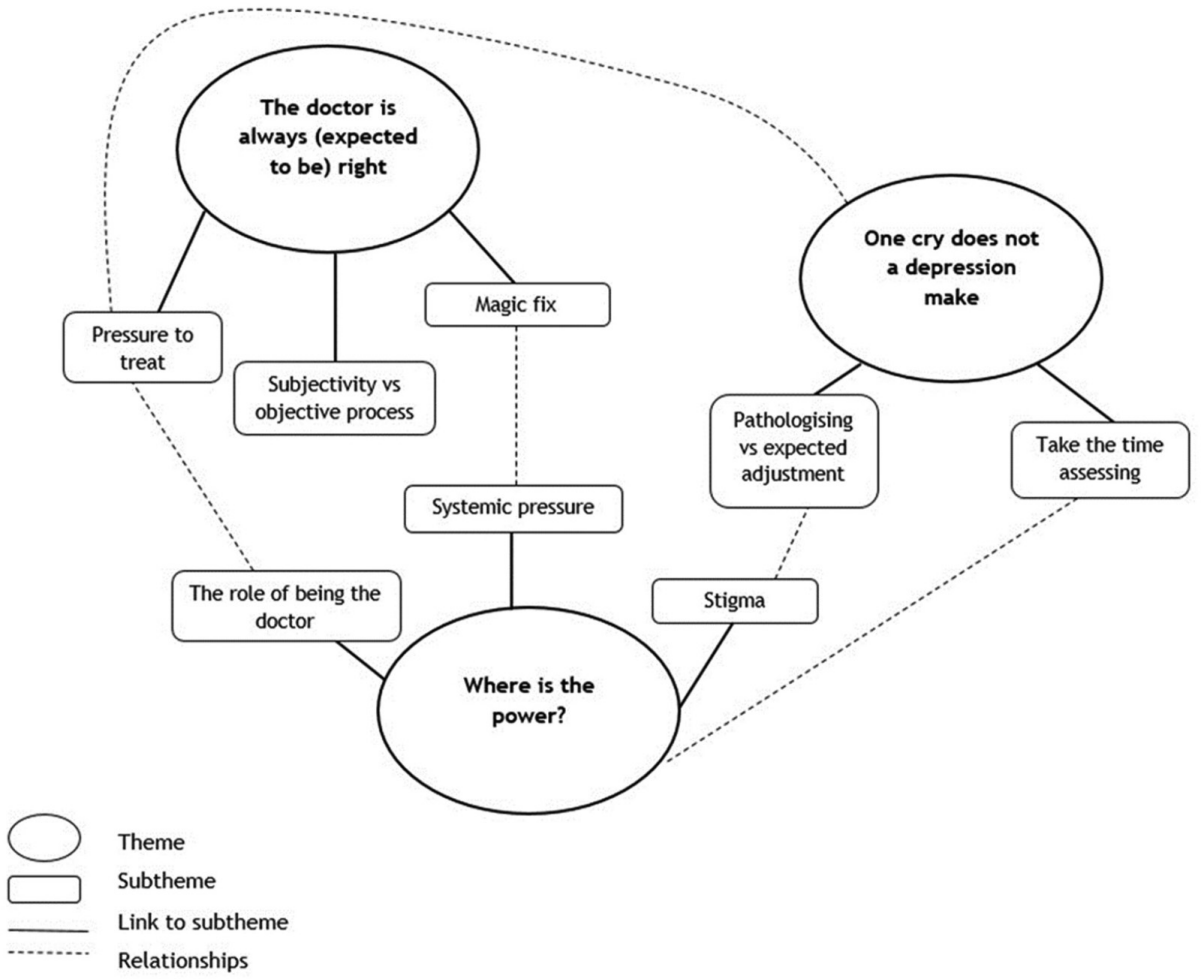
Medical professional interviews: implicit themes and how they relate to each other.

Latent (implicit) themes were labelled which relate to the assessment processes described above but give deeper insight into the implicit underlying meaning of the content. Figures 1 and 2 illustrate the latent themes and how they relate to each other.

Four latent themes were labelled in the interviews with clinical psychologists.

#### Feelings are scary: Pathologising and the pressure of dealing with emotions

Overall, clinical psychologists described the process of assessing mood in a severely impaired population to be challenging with a tendency of those working with the patient (the multidisciplinary team) to pathologise normal emotional responses. This pathologising may lead to unnecessary (according to the interviewees) referrals for mood assessment. Crying was seen as a normal reaction by the psychologists, not a presentation requiring professional psychology support, but rather emotional support from anyone in their environment. There is a view that as others may not want to deal with unpleasant emotions, they call in the psychologist instead.

*CP2: ‘…And I think we have quite a low tolerance of negative emotions. So people really want other people to stop crying, rather than thinking that maybe crying is okay, maybe we could help them with that and process it. And then maybe that'll be a different way to manage it. But I think professionals’ anxiety and professionals’ distress can be displaced, a bit of … it can be displaced, and then it's…people just want it to be gotten rid of’.*

CP2's response highlights a pattern of avoidance of emotional distress by other team members, which seems to speak to an intolerance of discomfort. Working within neurorehabilitation, most professionals are working to solve difficulties by retraining or compensating for loss of function which may make emotional difficulties more difficult to tolerate, resulting in increased referrals to psychologists who will ‘deal with that’.

*CP7: ‘I think there's probably an element of tolerating their own levels of distress. So the professionals’ distress in that. (Yeah). And it's not fixable in this, in the kind of looser sense of that word* [fixable]…’

This view of ‘referring’ emotions appears to lead to the psychologist to be external to the rest of the team, dealing with an intangible problem. There also appears to be a perceived increased pressure on the psychologist with this expectation to deal with the problem and offer a ‘quick fix’, which is not feasible, adding pressure to the complexity of assessing mood after severe brain injury.

#### Too many cooks: Managing multiple views and assessments

The psychologist is not the only one assessing mood; they work alongside medical professionals who perform their own assessments. Clinical psychologists discussed working with medical professionals and reported differing experiences which appeared to be based on personal relationships with the medical professional or their availability (permanent team doctor vs liaison psychiatrists). There were concerns about medical professionals’ experience and understanding of patients with severe cognitive and communication impairments:

*CP14: ‘I don't, I don't think I've ever seen a psychiatrist use supported communication with anyone, they don't… however much you say, ‘you know, do you want us to help, use some pictures?’ They don't…you know, they go in and just talk at the patient …’*

CP4 describes a good working relationship with a medical professional but alludes to an ‘either/or’ approach when it comes to medical and psychological assessment and intervention.

*CP4: ‘this is where we get the psychology versus consultant. And so I'm not, I'm definitely not anti-medication, I will put that out there first of all, but I really don't like it as a first line kind of approach… we discuss it in ward round…With the consultant…and our consultants…he's quite good at being led by psychology, which is quite nice. So he'll say, you know, “do you want to explore a bit more on that?”, “Yes, please! I'll come back to you in a few weeks”’.*

#### Uncertainty: Severe brain injury is different

Throughout the interviews, severe brain injury was differentiated from mild or moderate brain injury, meaning that it was discussed as being different and requiring a different approach and expert knowledge of the population. This occurred both by the nature of the interview protocol and by respondents highlighting that they would approach mood assessments differently with people with less severe impairment.

CP7: ‘*Absolutely! Yeah. Like, what are we [assessing]? What do we get? What's the gold standard for someone, you know, four weeks post traumatic brain injury, where they may have lost loved ones in an accident…’*

CP7's response alludes to severe brain injury perhaps not fitting with other understandings of what a normal reaction may be and how this may affect the use of measures in this population.

There seemed to be an unacknowledged sentiment that there is an insufficient evidence base in this area and that standardised measures are less trusted in this population, with some respondents expressing a lack of face validity.

*CP5: ‘Because I think a lot of the studies and the measures that are out there haven't been investigated with that particular population in mind…you might get things like false positive or false negative results… So that's always the risk that you take.* [so you should] *rely on multiple sources of information. And that measure, if you're using it, will be taken with a large grain of salt’.*

CP5's response acknowledges the usefulness of measures in other populations but raises concerns that they have not been validated in those with more severe impairments. The severity of cognitive impairment influenced views on the usefulness of measures for this population in particular.

Respondents reported being unsure if what they were doing was correct and being anxious about this, wanting a specific structure to increase their confidence in their results.

CP16: ‘*I suppose my experience is probably just like very kind of qualitative and probably like subjective, really, which I acknowledge isn't the most reliable or the most valid’.*

Interviewees found it reassuring when told that they had similar views to others, as seen in CP8 laughing and responding: (Laughs) ‘*That in itself is reassuring, because you can start to… as I was talking was my thinking…hmmm should I be doing this differently, maybe… I'm not sure. I'm interested to hear what other people do’*. Those with more experience were more comfortable discussing their uncertainty and relying less on measures more readily, citing their reliance on ‘instinct’ and ‘gut-feeling’.

CP6: ‘*And that gut instinct, you know, it's not based on nothing, is it? The gut instinct is based on experiences, based on training and it's based on kind of, you know, being around people who are depressed and anxious… but it's, it's very hard sometimes to kind of justify that’.*

It was notable when interviewees had not worked with the population group of interest that they struggled to fully appreciate difficulties. This could illustrate that specific experience and expert knowledge is needed to be able to identify where the issues may arise.

#### Opening a ‘Can of worms’: Limited time and resources

The process of assessing mood in this population was likened to ‘opening a can of worms’, both in the sense that it is complex, time consuming and involved and, as discussed in theme 1, the avoidance of dealing with something unpleasant in case it makes things worse.

CP2: ‘*Ooo it's scary, what if we open a can of worms?’… ‘I think what is quite interesting is that other staff members, so physiotherapists or maybe OTs, some of them are really good at it [dealing with emotions]. That's about them as individuals and what they're bringing to the job. They can be really good at holding the emotion, addressing it, identifying it. And then other people are terrible, and absolutely push it over to the side.’ … ‘its society isn’t it, we’re just not good at emotional stuff are we’.*

CP2 was discussing how other staff don’t necessarily have the training to deal with emotions and that as humans we are not all naturally comfortable with them, and that lack of training can lead to staff avoiding assessing mood.

Even within the role of the clinical psychologist the pressure of the system and amount of time and work involved in completing mood assessments in this population, may result in it being deprioritised:

CP22: *‘I guess one of the other things that comes up that's really challenging is that because people rarely are able to communicate, it's a really kind of time and resource intensive assessment, because you probably need to do quite a lot of behavioural assessment or kind of digging, like nursing, getting right lots of feedback we can do that you can then look through and charts, or getting lots of information from family members, or often we just don't have the resources to do it’.*

Three latent themes were identified from the interviews with the medical professionals.

#### The doctor is always (expected to be) right: Pressure to act

Multiple medical professionals spoke of the need to treat what was treatable – mood difficulties may respond to medication whereas cognitive difficulties and many other brain injury sequela probably will not, so there is pressure to treat. This overlapped with an implicit pressure placed on them by other team members when they had tried all other avenues and are hoping that medication will be a ‘magic fix’.

MP4: ‘*And so, yes, sometimes it is to treat or not to treat… but rarely in terms of medicine, you do nothing, because even by doing nothing…’ ‘It requires experience not to treat (Yeah), it requires experience to say, ‘Wait’, (yeah), so that itself is an activity’*

It was highlighted that the process of assessing mood can be quite subjective (based on experience, exposure and training) which can place additional pressure on the individual doctor to make a decision and appears to isolate them from the MDT, despite their diagnosis being partially based on collateral information.

MP5: ‘*…the way I felt about it was that it was super subjective. And that, basically, whoever is doing the assessment has already decided what they think, before they talk to patient anyway…And often, yeah, like assessing the patient is just kind of trying to back up what they already think’.*

#### One cry does not a depression make: Avoiding pathologising

The importance of consistency of symptoms was mentioned frequently – the need to observe symptoms in different scenarios, occurring frequently and causing difficulties to function before drawing conclusions regarding the presence of a mood disorder.

MP4: ‘*…and depression, we know that you know, a single swallow does not a summer make, and a single, single cry does not depression make, yeah, lots of depressives don't cry. So it's not so much about…it is the collection of symptoms, I think, and persisting over a period of time. Where the trajectory towards…the negatives, or no, no gains, or lack of gains when everything else should be happening’.*

Within this was an implicit theme of trying to avoid over-pathologising and over medicalising normal reaction to traumatic events. Taking the time to assess may result in delays in treatment which can place pressure on the doctor who is implicitly meant to ‘fix’ the issue.

MP6: *‘So it isn't that somehow or other this entity called depression is going to creep out of the woodwork and strike this person that you must, catch it while you can’… ‘Now, we all have mood. And sometimes our mood is better than worse. But most of us fluctuate within a reasonable level’.*

#### Where is the power? Power dynamics within the system

Medical professionals spoke of their assessment and treatment plans and implicitly relayed their awareness of power dynamics within the treatment environment. It was explicitly mentioned by MP3 who felt ‘*I think maybe because they [the patient] know that I'm a doctor, and I can do something, they might just say that I'm actually feeling pretty rubbish’.*

A less experienced consultant felt differently – MP2 reported ‘*I think like me asking questions on the ward round is often like not that helpful. I asked him on the ward round you know, how's your mood? And he's like “well, I am fine”, but I've got everyone telling me, like over the last three weeks, he's seems to be becoming more withdrawn and, you know, not engaging’.*

There were signs that the power dynamics between doctors may make it difficult to challenge decisions made by colleagues:

MP1: ‘*Because many times if you sit down and ask colleagues as to their rationale…because it's perceived as a challenge…yeah, can be an affront as well’.*

Difficulties with challenging more senior staff were also discussed:

MP5: ‘*So I would never really be the one that was like doing the proper mood assessment? It would always be Dr [name redacted] and [they] never used any scales…I'd do a little kind of preliminary type, like ‘how you feeling’. But I also knew that there was no point me doing a proper one because it would always ultimately be Dr [name redacted]'s decision’.*

Stigma of mental health disorders was acknowledged by interviewees.

MP4: ‘*It's also trying to avoid stigma on both ends, where if you've told the team that you're starting an antidepressant people think, ‘Oh, I don't think he's depressed’. But then they miss the bigger picture that this is actually, I'm not using it for its antidepressant properties… using it for behaviour modification or using it for… neuromodulation’*

This also implicitly introduced an additional power dynamic between experts on mental health and the rest of the multidisciplinary team, and between the patient and the medical system

MP8: ‘*The other difficulty other than the overlap kind of stuff, is that these conditions are still highly stigmatized. And many people don't want to talk about them so… or they think that they reveal some kind of weakness or insufficiency, which can be culturally and socially driven as well’.*

The pressure to work within a system can result in less than ideal practice and cause frustration, removing the power to act as they would want when completing assessments.

MP1: ‘*my approach did not always go well…with management…um…because I would favour multiple visits. As opposed to try to, you know, complete a referral…in some assessments you do, tidy up in one session’.*

## Discussion

This study explored the clinical practice of 23 clinical psychologists and nine medical professionals working with people who have cognitive and receptive communication difficulties after severe brain injury. No ‘gold standard’ approach to assessing mood in this population was evident. The construct of depression after severe brain injury was raised as being different from the construct in people with milder brain injuries and requiring a different approach to assessment. The suggested process from the interviews was a combination of collateral information, history, observations in multiple settings and over a period of time, and direct interview with patients. Although standardised measures were mentioned, these were adapted by the clinical psychologists and not routinely used by the medical professionals.

More experienced clinicians felt more confident relying less on standardised measures, consistent with previous findings that more experienced clinicians may be more comfortable with uncertainty.^17^ Interviewees expressed a shared experience of assessment being complex and raised concerns that this may be overlooked by professionals without experience of working with this population. Less experienced professionals may find it useful to be aware of pressure to ‘solve’ the issue of mood, when concerning symptoms may not be an issue with mood but arises from other consequences of the brain injury. A desire to avoid unpleasant emotions may lead to referrals to solve the ‘problem of distress’, which may be pathologising normal adjustment responses.

Four latent themes were identified in interviews with the clinical psychologists: 1. ‘Feelings are scary’; 2. ‘Too many cooks’: Managing multiple views and assessments; 3. ‘Uncertainty’: severe brain injury is different and 4. ‘Opening a “can of worms”’: limited time and resources. These themes highlight the complexities arising from the lack of an agreed approach in assessing mood in this population. Similar issues were raised in a study^18^ which found that adherence to guidelines when assessing mood after stroke was negatively impacted by issues such as concerns about the screening instruments, difficulties recognising mood symptoms after stroke, and uncertainty as to who should perform screening. The clinical psychologists in this study appear to apply their skills in formulation, alongside adapted standardised processes. These assessments can be complicated by power dynamics within teams, making a shared formulation or diagnosis challenging. An analysis of healthcare professionals^19^ showed that non-medical professionals reported having to negotiate their views to resolve interprofessional conflict, with a perception that medical assessments were prioritised. Guidance on multidisciplinary formulation of mood assessment after severe brain injury would be beneficial to support appropriate interprofessional assessment and treatment.

Three implicit themes were identified in medical professional interviews. ‘Where is the power?’, indicating awareness of hierarchical issues and power imbalances and ‘one cry does not a depression make’ illustrating the concerns about pathologising emotions. The ‘doctor is always (expected to be) right’ theme highlighted expectations of doctors to ‘fix’ the issues by using medication, suggesting a medicalised view of the problem. This need for a ‘magic fix’ using medication may relate to the complexity in treating patients with severe cognitive and receptive communication impairments and a desire to ameliorate the difficulties.

A significant concern was raised regarding the follow up and side effects of antidepressants and this population's inability to reliably self-report, concerns that are supported by findings on patient reported unpleasant side-effects from antidepressants.^20^ Additionally, a recent systematic review^21^ suggested that current guidelines do not adequately address the incidence of antidepressant withdrawal and raised concerns of misdiagnosing withdrawal as recurrence. Experts working with this population should give clear guidance to those that may be less expert on when and how to reduce and stop antidepressants.

Strengths of this study include consistency of themes indicating a degree of shared experience between different professionals. Participants had varying levels of qualification and experiences, which made the finding of overlapping themes encouraging. Limitations of the study include that it was conducted and analysed by one person (AR), though efforts were made to reduce bias. The small number of medical professionals recruited may be indicative of a highly specialist patient population and thus fewer appropriate candidates for interview, though again there was consistency of views within the group. Additionally, it would be beneficial to ensure that the views of other frontline staff involved in assessing mood and caring for this patient population are incorporated in future research given that the views of healthcare professionals who are caring full time for people with brain injury may differ from specialist mental health professionals.

The results of these interviews highlight the need for a different approach to assessing mood when cognitive and receptive communication impairments are present after severe brain injury. Considering the small number of experts in this field, the insights gained here are important in shedding light on current clinical practice of clinical psychologists and medical professionals working in this field. These results may also be useful in informing the practice of others assessing mood in people with cognitive and receptive communication impairments after severe acquired brain injury. Future work focussed on developing a consensus would benefit this clinical population and focus groups were completed by the authors to explore this (manuscript under peer review).

**Clinical messages**

- There is no gold standard approach to assessing mood in people with severe cognitive and receptive communication deficits after brain injury and self-report measures are not suitable.
- Over-pathologising of expressions of emotional distress in the presence of severe cognitive impairment should be avoided.
- To assess this patient population, clinicians should ask for views from staff and family, complete observations, have in-the-moment patient assessments, and complete assessments over time.

## Supplemental Material

sj-docx-1-cre-10.1177_02692155241278289 - Supplemental material for Assessment of mood after severe acquired brain injury: Interviews with UK clinical psychologists and medical professionals Supplemental material, sj-docx-1-cre-10.1177_02692155241278289 for Assessment of mood after severe acquired brain injury: Interviews with UK clinical psychologists and medical professionals by Alexandra E Rose, Breda Cullen and Sarah Crawford, Jonathan J Evans in Clinical Rehabilitation
