# Supplementary material for: Assessment of mood after severe acquired brain injury: Interviews with UK clinical psychologists and medical professionals
Source: Clin Rehabil. 2024 Sep 10;38(11):1521–33. doi: 10.1177/02692155241278289 (PMC11528957; doi:10.1177/02692155241278289)
Supplement: sj-docx-1-cre-10.1177_02692155241278289 - Supplemental material for Assessment of mood after severe acquired brain injury: Interviews with UK clinical psychologists and medical professionals [file sj-docx-1-cre-10.1177_02692155241278289.docx]

## Supplementary Materials

Supplementary Table 1: Clinical psychologists (CP) experience and standardised mood measure usage

| **Interview** | **Experience with population of interest** | **Uses measures** | **Assessment measures used** | **Adapts Measures** | **Adapts scores of measures** |
| --- | --- | --- | --- | --- | --- |
| CP1 | Yes | Yes | HADS, GAD7, BASDEC, PHQ (SDSS, SADQ in SABI) | Yes | Yes |
| CP2 | Yes | Yes | BAI, HADS (Not in SABI) | Yes | Yes |
| CP3 | Yes | Yes | HADS (Not in SABI) | Yes | Yes |
| CP4 | Yes | Yes | HADs, BDI, PHQ (SDSS, DISCs in SABI). | Yes | Yes |
| CP5 | Yes | Yes | HADS -adjusted (Not in SABI) | Yes | Yes |
| CP6 | Yes | Yes | SDSS, Yale and BASDEC (No BASDEC in SABI) | Yes | Yes |
| CP7 | Yes | Yes | PHQ9 (Not in SABI) | Yes | Yes |
| CP8 | Yes | Yes | DISCs, BDI, HADS (Not in SABI) | Yes | Yes |
| CP9 | Yes | Yes | VASES, BDI (Adjusted) | Yes | Yes |
| CP10 | Yes | Yes | SADQ, HADS (Not in SABI) | Yes | Yes |
| CP11 | Yes | Yes | SADQ | Yes | Yes |
| CP12 | Yes | Yes | HADS (SADQ, DISCs in SABI) | Yes | Yes |
| CP13 | Yes | Yes | HADS, PHQ, GAD (SADQ in SABI) | Yes | Yes |
| CP14 | Yes | Yes | DISCs (Not in SABI) | Yes | Yes |
| CP15 | Yes | Yes | PHQ9, GAD7, BDI, HADS, DASS21. (DISCs in SABI) | Yes | Yes |
| CP16 | Yes | Yes | HADS, BDI, BAI (Smiley faces in SABI) | Yes | Yes |
| CP17 | No | Yes | DASS21 | No | Yes |
| CP18 | Yes | Yes | PHQ9, GAD (SADQ10 in SABI) | Yes | Yes |
| CP19 | Yes | Yes | DISCs, BDI, HADS- adjusted. (SADQ in SABI) | Yes | Yes |
| CP20 | No | Yes | PHQ9, GAD7, CORE10, BASDEC, SADQH10, DISCs (Adjusted in SABI) | Yes | No |
| CP21 | Yes | Yes | CORE10, DISCs, PHQ9, GAD7, HADS (Not in SABI) | Yes | Yes |
| CP22 | Yes | Yes | Faces and observer rated measures (Not in SABI) | Yes | Yes |
| CP23 | Yes | Yes | HADS (Not in SABI) | Yes | No |

BAI=Beck Anxiety Inventory, BASDEC=Brief Assessment Schedule Depression Cards, BDI=Beck Depression Inventory, CORE10=Clinical Outcomes in Routine Examination, DASS21=Depression, Anxiety, Stress Scale, DISCs=Depression Intensity Scale Circles, GAD = Generalised anxiety disorder assessment, HADS== Hospital anxiety and depression scale, PHQ=Patient Health Questionnaire, SDSS=Signs of depression Screening Scale, SADQ,=Stroke Aphasia Depression Questionnaire, SABI=Severe Acquired Brain Injury, VASES = Visual analogue self-esteem scale. , Yale = Yale question.

Supplementary Table 2: Medical professionals (MP) experience and standardised mood measure usage

| **Interview** | **Experience with population of interest** | **Uses measures** | **Adapts Measures** | **Adapts scores of measures** | **Measures used** |
| --- | --- | --- | --- | --- | --- |
| MP1 | Minimal | No | N/A | N/A | N/A |
| MP2 | Yes | No | N/A | N/A | N/A |
| MP3 | Yes | No | N/A | N/A | N/A |
| MP4 | Yes | No | N/A | N/A | N/A |
| MP5 | Yes | No | N/A | N/A | N/A |
| MP6 | Yes | No | N/A | N/A | N/A |
| MP7 | Yes | No | N/A | N/A | N/A |
| MP8 | Yes | Yes | No | Yes | PHQ, MADRS, VASES, GAD, GAF |
| MP9 | Yes | Yes | No | Yes | EBIQ, MMPI, HADS |

EBIQ= European brain injury questionnaire, GAD= Generalised anxiety disorder assessment, GAF= Global assessment of functioning, HADS= Hospital anxiety and depression scale, MADRS= Montgomery-Asberg depression rating scale, MMPI= Minnesota multiphasic personality inventory, N/A= not applicable, PHQ= Patient health questionnaire, VASES= Visual analogue self-esteem scale.

Supplementary Table 3: Clinical psychologists’ semantic themes

| **Theme** | **Sub themes** | **Description of theme** | **Quotes from interviews** |
| --- | --- | --- | --- |
| 1. **Conceptualisation** | - The construct of depression - Normal adjustment | - Depression may look different after brain injury - Emotions may be normal and not related to diagnostic criteria | *CP1: “the main difficulty that we have with mood in brain injury is that the constructs are weak. And actually, I think that even if we go and think about the constructs that I've talked... spoken about in the neuropsych formulation, like the idea of cognition, behaviour, and emotion, being separate entities, is purely constructed through ourselves because it's easier to think of things in those types of categories. But actually, they're all intertwined…. Now, the difficulty with that is that, for me, I feel that to fully understand that, to understand it to the best of our abilities, you really have had to have a lot of training, because you really need to be thinking about how all of these different areas interact. So in* *order to understand mood, you need to understand all of the other complex variables that are that are at play."*  *CP8: “I suppose it depends on what you think depression is? [Pause] I don't know. I mean, what would Beck say? [Laughs] Good old Beck would say his triangle, wouldn't he….Like self, world, others and thinking about the future and all sorts of things like that. And I think maybe some people in this population don't have a clear sense of self anymore or don't have a clear sense of what the future is…. [They] can't sort of think back and… maybe living very much in the moment. So it's tricky, because you can't really get at it anyway. Like, you're using measures and observations and what people say….So if someone can't tell you very easily, that's quite difficult to know what's happening. I don't know if they are the same thing or if it's [long pause] Hmm. Tricky.”* |
| 1. **Assessment** | - Process - Symptoms - Reliability | - The recognition that mood needs to be assessed - Symptoms to be monitored include sleep, appetite, tearfulness, interaction with others and engagement - Responses may not be reliable, standardised measures may not be reliable. | *CP8: “So probably something about change over time. So is it kind of a... [Pause]…and consistencies? Like, is it a one off that somebody was crying? Or is it that somebody is crying every day, and not doing much at all when they were before? Or you believe that able to? Or is there a behaviour thing that’s showing up a lot?”*  *CP12: “I think, especially when some services may be a little bit kind of stretched…or the psychology provision is limited, [pause] I think the obvious kind of tipping point for us is where the kind of questions or concerns about mood are raised in terms of a person's ability to engage in their rehabilitation. So maybe where their level of motivation doesn't seem particularly high, or the physios are getting kind of something in the gym but it's a struggle to get them engaged or involved with different sort of exercises, or the OTs are struggling to get them to do the more functional tasks and the person is... is distressed or worried or anxious? That probably is a fairly obvious kind of point where we [psychology] could probably get more engaged and more involved and be asked to kind of assess those things.”* |
| 1. **The person with severe brain injury** | - Person-centred - Culture - Cognition - Communication | - Bespoke and person centred approaches are beneficial - Consider the role of culture - Understanding cognitive impairment is necessary - Adapting to communication needs is necessary | *CP5: “Yeah, and it's [the formulation] really individualised… And it's all about seeing the person behind the brain injury and seeing the person who was there before, what we see now and why are we seeing what we're seeing now. So, more often than not, that is helpful to share that [formulation] with the treating team as well. But sometimes we might, you know, take out certain bits if we felt that it was very personal or didn't necessarily contribute to the impact of their care.”*  *CP17: “I think a big factor is cultural differences. So for a lot of ethnic…. ethnic communities, they're not actually... I guess…You know, they describe emotional pain and they describe it in physical terms...so somaticize the description of their mood. Yeah. That can be a challenge.”* |
| 1. **The assessor (Clinical Psychologist)** | - Confidence - Experience - Instinct | - Clinical opinion requires confidence (including to not use standardised measures) - The more you experience severe brain injury, the more you are able to recognise the complexities - Sometimes you can have an instinct that mood needs to be addressed/may be an issue | *CP19: “I think, for me, because… I think I ask trainees or assistants to assess mood…. I think it [mood measures] also gives them an anchor for what they're doing because sometimes it could be feeling like it's a bit of a safety net to know what they could ask. Whereas I probably… probably lean on it [mood measures] less myself, but might get someone to say oh can you do that and just see if there's anything that comes out of it that might indicate we need to look into a bit more.”*  *CP3: “And it's like, well, in your mind, you kind of think, well, I need to do an assessment and kind of brings about this image of like a formal assessment, and I think maybe it…I think it takes quite a lot of confidence to be able to assess somebody's presentation without a pen and paper, sometimes I think, maybe particularly in the NHS, we're not really encouraged to do things informally, if that makes sense, or it is deemed to be informal, if it's not a pen and paper. Yeah, I think…there is something to that, that it's formalising and the tick boxing exercise. Yeah. Also, I wonder whether there's there just isn't anything available, which may suit our, our population. And so it's kind of like the closest thing we've got. So it could perhaps…it's a confidence thing, and it's very much, it's like a framework* *for us to base our questions on for example.”* |
| 1. **The system** | - The role of the psychologist - Medical views - Views of others | - Psychologists are expected to deal with distress and difficult situations - Pathologising of emotions and medicalising adjustment or normal reactions - Others may project their views onto those with severe ABI. | *CP14: “No. Not really, I mean, I do them [mood measures] just so I can tick the SSNAP [The Sentinel Stroke National Audit Programme] box …would have had their mood assessed… but I don't, I don't actually feel it adds anything to my clinical impression, I, maybe it's arrogance on my behalf, but I would always trust my clinical judgment over and above the mood assessment.”*  *CP10: “My role with this is whether this is a normal adjustment response versus, actually, this is someone who is developing quite adept insight and it is beginning to have a severe and enduring impact on their mood, together with... and if they developed self-harm ideation or a self-harm plan that they might have had… [Pause]… So within our unit, we have a sessional input from psychiatry and it will be liaised across there....we have reasonably good relationships with neuropsychiatry here and rehab medicine…. It's very much… try to reach a view and inform the day to day patient management.”*  *CP7: “… because often the doctors that have kind of come on to the ward, may have been in other positions where a PHQ-9 is used as a reliable indicator of whether someone is depressed or not. And so if they have a score above a certain degree, well, then maybe an antidepressant would be indicated. And actually, I'm having those kinds of conversations to shift that narrative slightly of going well, actually, this is a transient process. It's not something that is linear. And I think that's the function for the medics, particularly around a questionnaire measure is about the score, and therefore* |

Supplementary Figure 1: Semantic themes regarding assessment process by clinical psychologists


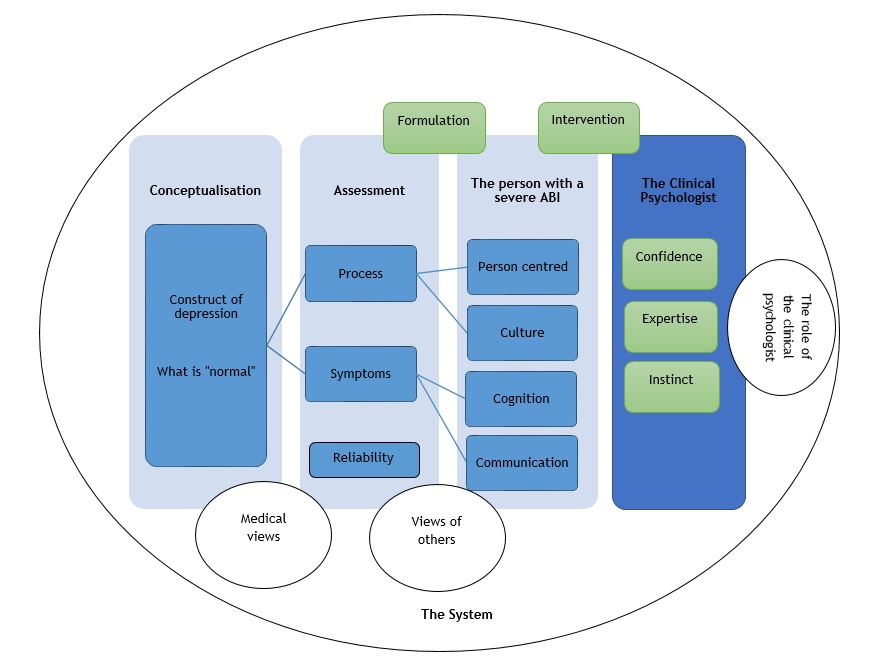


Supplementary Table 4: Medical professionals’ semantic themes

| **Theme** | **Sub themes** | **Description of theme** | **Quotes from interviews** |
| --- | --- | --- | --- |
| 1. **Conceptualisation** | - Depression after ABI - Neurotransmitters | - Differing views on whether depression after ABI is different to other presentations of depression - Depression and neurotransmitter disruption | MP6: *“How I use them [terms] will depend on the context, so if I am discussing it with us sort of medically orientated and not doctor orientated but health orientated people, I would tend to refer to distress or low mood, because as soon as you say depression they [the team] say ‘Ah antidepressants’. So we, we as a sort of collected health service people get terribly attached to labels and immediately jump to a conclusion”*  MP4: *“I mean, I don't think any of the DSM criteria apply to this end of the spectrum, really, of diagnosing clinical depression, or for that matter, assessing it, you know”*  MP6: *“Rather than think that depression is somehow categorically different (Yes) in someone with a brain injury, it is possibly a different proportion of things. (Yeah), but it isn't a Category differences, just what the factors are difference.”*  MP8: *“I think it's the same illness. But I think you get to it in different ways”…. “And there are obviously biological, psychological, social factors, which impacts upon mood. And the endpoint, the end organ, which is responding to all of those biological, psychological, social factors is the brain.”*  MP9: *“So I often talk about it being a change in the chemical balance between the sort of activating and lowering calming chemicals in the brain.”* |
| 1. **Assessment** | - Organic causes - Assessment Process - Symptoms - Formulation/ differential diagnosis | - Ruling out organic causes for behaviours. - How they approach assessment - Symptoms that indicate mood may be difficult to identify - create formulation/rule our other diagnoses | MP7: *“And when you think about the reasons why people deteriorate after brain injury, after hydrocephalus, the most common thing is depression. And then later on, the most common thing is dementia. Depression comes along way up the line (pause) if they're deteriorating, because people with brain injury get better and then plateau (pause) if somebody's deteriorating and they haven't got hydrocephalus, you can pretty much put a stamp on it and say they might be depressed”; thus highlighting that ruling our organic causes for symptoms is important, which was echoed by others.”*  MP5: “*Rule out any organic causes like infections. So do some Bloods (pause) and if I have any suspicion of hydrocephalus, maybe consider ordering a CT scan”… “Examination, over time, obs, bloods usual investigation, urine dips, ECG, X-rays”*  MP9: *“I'd certainly look for any biochemical explanation. So again, if people have got low cortisol, or in fact high cortisol can be associated with depression but also with post injury hypopituitarism of any sort, so, you know, hypothyroidism, I certainly if there's a significant impression that somebody's presenting with a, you know, significantly low mood, I would certainly check the pituitary function after a head injury.”*  MP3: *“They could be quite apathetic because of the frontal lobe injuries or injuries to the basal ganglia. I find that people who had some sort of hydrocephalus at some stage or still have some ongoing increased ranges usually appear like they are depressed. But it may not be the true depression.”*  MP6: “In terms of assessment, I don't use any standardized questionnaires. I think that they have limited use, you can be misled by a survey of depression, but probably not much other supportive evidence. And you can also have someone who looks very depressed but does quite well on the questionnaires. So I prefer to have an overall clinical judgment.”  MP9: “*I think, and you also get people who are very restless and agitated with even more signs, if you like, than somebody [who] hasn't got a brain injury. But that doesn't mean they're necessarily depressed, you kind of have to reinterpret, whatever you're seeing, as you said, it might be less obvious, it might look much more overt”.*  MP4: “*we have a collection of signs symptoms everyone's putting into the pot and saying that, you know, on a balance of probabilities more likely than not this behavioural syndrome, we're not talking about whether it is a diagnosis of depression or brain injury, we're saying on balance of probabilities, the intervention [for low mood] is likely to be beneficial*.”  MP8: “S*o if somebody is suicidal, that's not associated with brain injury, if somebody's hopeless, that's not associated with brain injury, if somebody's guilty, low self-esteem, not enjoying anything, those are my kind of big six, including low mood, that I would say, are better at differentiating between mood and brain injury related kind of presentations.*  MP8: *“In terms of that diagnostic formulation work, is about making a...balance of probabilities decision about what a symptom represents in terms of underlying psychopathology, whether that's neuropathology, psychopathology, cognitive, emotional, whatever it might be, and saying, well, on balance, I think that this person is presenting with predominately ‘negative type’ symptoms, like apathy, a motivation and poor initiation, that those are more related to mood than they might be to underlying brain injury. And, therefore, that's how we make a decision about treating it or not...um being whether it's a reversible factor that is treatable.”* |
| 1. **The person with severe brain injury** | - Discrimination - Cognition - Communication | - The presence of a brain injury does not prevent normal human emotional reactions - Poor historians due to memory impairments - Communication impairments make assessment challenging | MP3: *“very poor cognition is an issue as well. You never know. You have to assess the cognition and can't pick what's going on in there”…. “They may not be able to express what they are feeling or what their situation is inside”.*  MP4: “*But do they have the necessary cognition and therefore an ability to generate behaviours that tell you that somebody has low mood or is sad or even the other way around.”*  MP9: *“so then the next thing I would do is try and understand how people have been communicating with that person. So, again, trying to understand from family or team or if, ideally, if they've seen a speech therapist, you know, are they able to indicate yeses, and noes, how much would they understand? Is it going to be better to use some pictures?”* |
| 1. **Treatment options** | - Medication - Non-pharmacological interventions | - Reviewing medication choices and side effects - Not using medication as first line of treatment | MP3: *“So sometimes I think you have to try it. (Yeah). Sometimes you just need to think about giving an antidepressant not because people are depressed. Just to give them that boost. (Yeah). So they can just get their brain engaged. (Yeah). Because at the end how does an antidepressant work? It works on the neurotransmitter system.”*  MP8: *“Yeah. So I certainly I,...that's what I was kind of alluding to earlier on that even without reaching caseness for a mood disorder, I think there is some suggestion that there are improvements in brain injury related phenomena with some of these medications, which on face value you might expect, because...um... and the medicines as well, the other problem with it all is that medicines cross domains, too, so they don't.... So you might be using a stimulant to improve somebody's inattention. But that also has a mood related impact.”*  MP1: *“And sometimes also, without thinking, I think we just throw medication, forgive my indiscretion, that we have at people hoping to see what effect Yeah, things not happening quickly, then they add on a set of haloperidol, Risperidone… mix and match. And we don't pay attention to likely side effects as well”.*  MP5: “*How to ask about side effects? Yes, we do. I mean, in terms of checking, we did regular ECG and blood tests. And then in terms of symptoms, normally check with like the nurses on how they're responding. Often like the side effects are actually, yeah, like you said, there's kind of what you want it to act in like balancing out the person.”*  MP1: *“But the antidepressant might take weeks and months to work, what about the other non-pharmacological, psychological essence language and as best as possible, culturally appropriate? (Yeah). Putting all those things together. So it's not just medication.”*  MP3: *“So there is an organic element. There is a functional element to the...the functioning of the brain. (Yeah. Yeah) and they don't have the resources to do other things. (Yeah). So you can't do talking therapies much. You can't do I don't know... mindfulness exercises when they are feeling anxious or hopeless or panicky. So you have to find the right thing.”* |
| 1. **The assessor** | - Experience - Training | - Learn on the job - Concerns about community follow up - Not routinely trained in assessing people with cognitive and communication impairments | MP2: *“I think that like the really distressed patients, I think are the ones that we tend to speak to neuro psychiatry quite early on about because I think, where I might have like, one idea of, like…maybe a different medication might be appropriate.”*  MP5: *“I think the GPs, (pause) probably just doing repeat prescriptions every time. And I mean, there's no way that a GP can manage the medications, considering they got like seven minutes to see a patient. And in seven minutes with those patients, you can barely like, say hello. Yeah. And that, yeah, it's so complex and if I was the GP, I'd be like, okay, wow, a neuro rehabilitation consultant has prescribed this. I'm going to continue it because they know better than me.”*  MP9: “*I think they can get left on things I think it doesn't get reviewed properly (Yeah), I do think it's not commonly...you know it's still a challenge to remember everything. And sometimes people might not check for the pituitary dysfunction, or might not check for changes in CSF for example, hydrocephalus”.*  MP3: *“…brain injury patients waiting in an acute, you know, like a general medical ward, you know, in a gastro ward. You know, who is going to understand the brain injury and assess their mood or behaviour or anything, they just get the nursing until they come to rehabilitation and then by the time they come to attention, maybe they are in a very severe depression.”*  MP1: *“So on the one hand, I felt for… a liaison service attached to a general hospital that happened to have a major trauma unit, we did not really have much, if any training in terms of assessing patients with cognitive sequelae follow a TBI. Most time it was usually subjective (mmhmm) and I often felt that was either very rushed or kind of simplistic reductionist-type diagnoses without necessarily giving thought… to …presentations, differentials even.”*  MP5: *“Yeah, because you have we don't, you don't get any training? Yeah, about people with brain injuries and medical school, in any medical rotations... So yeah, I didn't know anything about how to communicate with patients.”… “And to be honest, I didn't really get any training anyway, either. So just kind of learn everything on the job.”* |


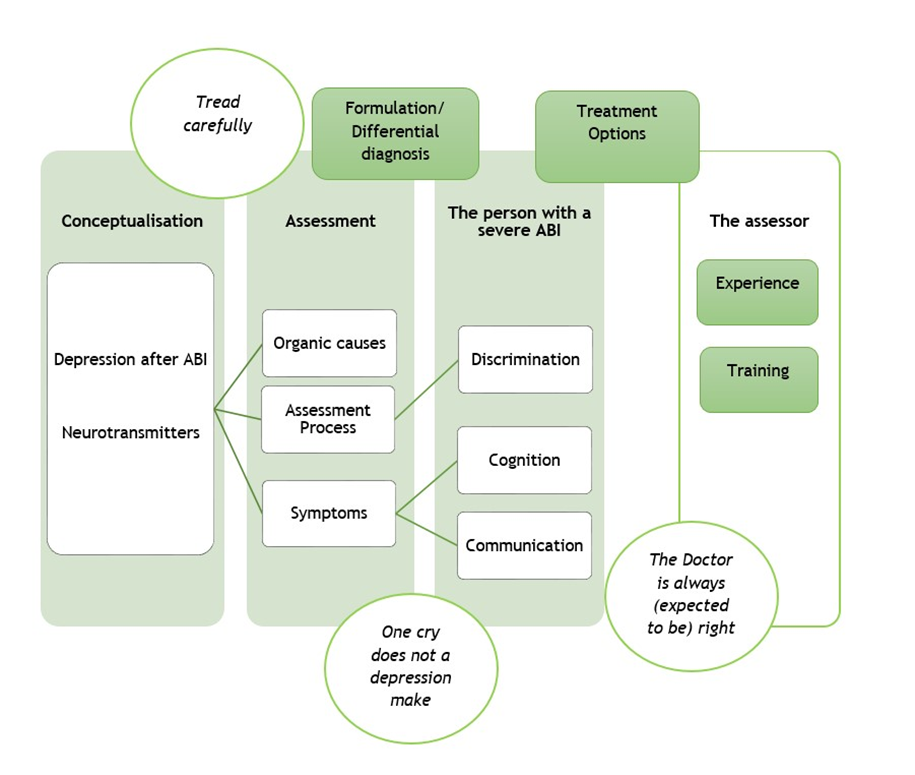
Supplementary Figure 2: Semantic themes regarding assessment process by medical professionals
